# Immune Response to *Mycobacterium tuberculosis*: A Narrative Review

**DOI:** 10.3389/fped.2019.00350

**Published:** 2019-08-27

**Authors:** Maurizio de Martino, Lorenzo Lodi, Luisa Galli, Elena Chiappini

**Affiliations:** Department of Health Sciences, University of Florence, Florence, Italy

**Keywords:** *Mycobacterium tuberculosis*, tuberculosis, children, immune response, immunity, macrophage, adaptive immunity, granuloma

## Abstract

The encounter between *Mycobacterium tuberculosis* (Mtb) and the host leads to a complex and multifaceted immune response possibly resulting in latent infection, tubercular disease or to the complete clearance of the pathogen. Macrophages and CD4^+^ T lymphocytes, together with granuloma formation, are traditionally considered the pillars of immune defense against Mtb and their role stands out clearly. However, there is no component of the immune system that does not take part in the response to this pathogen. On the other side, Mtb displays a complex artillery of immune-escaping mechanisms capable of responding in an equally varied manner. In addition, the role of each cellular line has become discussed and uncertain further than ever before. Each defense mechanism is based on a subtle balance that, if altered, can lean to one side to favor Mtb proliferation, resulting in disease progression and on the other to the host tissue damage by the immune system itself. Through a brief and complete overview of the role of each cell type involved in the Mtb response, we aimed to highlight the main literature reviews and the most relevant studies in order to facilitate the approach to such a complex and changeable topic. In conclusion, this narrative mini-review summarizes the various immunologic mechanisms which modulate the individual ability to fight Mtb infection taking in account the major host and pathogen determinants in the susceptibility to tuberculosis.

## Introduction

Tuberculosis (TB), caused by *Mycobacterium tuberculosis* (Mtb) infection, was among the top 10 causes of death worldwide in 2017 with about 1.5 million registered deceases (1). Mtb was responsible for approximately 10.0 million incident cases of TB disease with 10% of these occurring among children (1). One to five bacilli may suffice to transmit the infection by air (2). When inhaled, Mtb encounters a first line of defense consisting of airway epithelial cells (AECs) and “professional” phagocytes (neutrophils, monocytes and dendritic cells) (3, 4). If this first line succeeds in eliminating the Mtb rapidly, the infection aborts (5). Otherwise, phagocytes are infected and the Mtb reproduces inside the cells, initially causing few, if any, clinical manifestations (5). The establishment of the infection, the development of active TB (ATB) rather than latent TB infection (LTBI) and the eventual evolution of LTBI to ATB depends on the complex relation between bacterial and host factors.

The aim of this narrative minireview is to give a hint of the complexity of the above-mentioned determinants and to briefly summarize the major defense mechanisms of innate and adaptive immunity against Mtb outlining the role of the different cell populations and their complex interplay.

## Methods

In order to perform a narrative review of the available literature, we searched the PubMed database from April 2014 through April 2019, using the following key words: “immune,” “immunity,” “tuberculosis,” “*Mycobacterium tuberculosis*.” Subsequently, for each topic, specific key words (“susceptibility,” “resistance,” “virulence,” “airway epithelial cell,” “macrophage,” “neutrophil,” “dendritic cell,” “natural killer,” “mast cell,” “complement,” “CD4,” “CD8,” “humoral,” “antibody,” and “granuloma”) were associated with the word “tuberculosis” in order to access proper specific literature. The search and the selection process were not systematic. Articles were limited to English language and full text availability, and they were excluded if they were redundant or not pertinent. References of all relevant articles were also evaluated, and studies published previously than 2014 were cited if considered relevant. Results were critically summarized in the following paragraphs: (1) “host and bacterial determinants in human tuberculosis,” (2) “innate immune response against *Mycobacterium tuberculosis*,” and (3) “adaptive immune response against *Mycobacterium tuberculosis*.”

## Host and Bacterial Determinants in Human Tuberculosis

Several epidemiological models of family members who have long shared the bedroom with subjects with ATB, sailors who lived in confined spaces with subjects with open TB and extensive case studies of South African miners and Norwegian or American students, have clearly demonstrated that 5 to 20% of those who meet subjects with ATB do not become infected (resilient individuals or *resisters*), or become infected only transiently and then get rid of the infection (early sterilization or *early clearance*) (6). An individual can be defined resilient if after close and prolonged contact with the index case shows simultaneous negativity of the skin reactivity test and of the IFN-γ release assay (IGRA) which persists for at least 1 year. Studies carried out on siblings have shown that Mtb resilience is more frequent between two siblings than between two unrelated subjects, suggesting the role of genetics in the development of Mtb resilience (7). Genome wide linkage analysis detected several loci like *2q21-2q24, 5p13-5q22*, and the *TST1* on *11p14* associated with the resilient phenotype (8, 9).

On the other side, the study of TB susceptibility, has shed light onto various components of immunity to mycobacteria in humans. Different genetic polymorphisms which modulate the host immune response in favor of TB infection and disease progression have been identified in human leukocyte antigens (HLA), toll like receptors (TLR), vitamin D receptors (VDR), cytokines with their receptors and many other functional immune components (10, 11). Moreover, mendelian susceptibilities to mycobacterial disease (MSMD) have been identified as clinical conditions with selective susceptibility to poorly virulent mycobacteria in the absence of patent immunodeficiency (12). Since 1996, 11 genes which underlie 21 different genetic disorders related to interferon (IFN)-γ immunity and responsible for MSMD have been identified (12).

Furthermore, transcriptomic studies have described a TB signature of neutrophil-driven IFN-inducible genes in ATB, including IFN-γ but also type I IFNs, reflecting disease extension and response to treatment and highlighting the previously under-appreciated role of IFNαβ signaling in TB pathogenesis (4, 13, 14).

Beyond host factors, bacterial virulence constitutes the other major player when evaluating the risk of TB infection. Virulence is not merely limited to bacterial strain or burden in respiratory secretion but takes into account the differential Mtb gene expression in the different phases of infection. Mtb lacks classical virulence factors such as toxins and its immune-escaping ability depends on the modulation of lipid metabolism, metal-transporter proteins, protease, proteins inhibiting the antimicrobial effectors of macrophages (MΦs) and many others (15).

The study of immune response in resilient and susceptible individuals, together with bacterial factors, has offered fundamental information for the understanding of TB immunology suggesting potential improvements in diagnostic and therapeutic approaches (Table 1).

**Table 1:** Key literature of the present review.

**Paragraph**	**Topic**	**Major references**
Host and bacterial determinants in human tuberculosis	Host Genetic polymorphism	-**Harishankar et al**. **(****10****)** - Casanova and Abel (7) - Cobat et al. (8) - Stein et al. (9) - van Tong et al. (11)
	MSMD	-**Rosain et al**. **(****12****)**
	Transcriptomic studies	-**Berry et al**. **(****13****)** - Blankley et al. (14)
	Bacterial Virulence factors	- **Forrellad et al**. **(****15****)**
Innate immune response against *Mycobacterium tuberculosis*	AECs	- **Li et al**. **(****16****)** - Harriff et al. (17)
	Macrophages	- **Queval et al**. **(****18****)** - Lerner et al. (19) - Yuk et al. (20) - Gröschel et al. (21) - Bustamante et al. (22) - Sia et al. (23) - Sun et al. (24) - Neyrolles et al. (25) - Botella et al. (26)
	Neutrophils	- **Kroon et al**. **(****27****)** - Lowe et al. (28) - Tan et al. (29) - Zhang et al. (30)
	Dendritic cells	- **Mihret** **(****31****)** - Khan et al. (32) - Wu et al. (33) - Balboa et al. (34) - Georgieva et al. (35) - Velasquez et al. (36) - Ehlers (37)
	NK cells	- **Esin and Batoni** **(****38****)** - Arora et al. (39) - Zhang et al. (40)
	Mast Cells	- **Garcia-Rodriguez et al**. **(****41****)** - Carlos et al. (42)
	Complement	- **Lubbers et al**. **(****43****)** - Cai et al. (44)
Adaptive immune response against *Mycobacterium tuberculosis*	CD4^+^ T lymphocytes	- **Cooper** **(****45****)** - Sia et al. (46) - Domingo-Gonzalez et al. (47) - Parkash et al. (48) - Sallin et al. (49)
	CD8^+^ T lymphocytes	- **Lin and Flynn** **(****50****)** - Canaday et al. (51) - Oddo et al. (52)
	Humoral immunity	- **Kozakiewicz et al**. **(****53****)** - Jacobs et al. (54) - Glatman-Freedman and Casadevall (55) - Lu et al. (56)
	Granuloma	- **Ramakrishnan** **(****57****)** - Russell (58) - Reece and Kaufmann (59) - Refai et al. (60) - Martinot (61) - Russell et al. (62)

*The major references are grouped basing on the different topics addressed in each paragraph. The most relevant and comprehensive review or study for each topic is highlighted in bold. MSMD, Mendelian Susceptibility to Mycobacterial Disease; AEC, Airway Epithelial Cell; NK, Natural Killer cells*.

## Innate Immune Response Against *Mycobacterium tuberculosis*

The significance of innate immunity in the defense against Mtb stands out clearly as we consider the MSMD where a disruption of the innate axis leads to dramatic, life-threatening clinical presentation of TB (12).

MΦ, neutrophils, dendritic cells (DCs), natural killer cells (NK), mast cells and complement are the major players of innate immunity. On the other hand, AECs also contribute to the defense attempt against Mtb and could be considered as innate immunity components (Table 1).

### Airway Epithelial Cells

AECs are the first cells to come in contact with Mtb. Beyond their major role as physical barriers, they display several immunological functions albeit being traditionally considered as “non-professional” immune cells. Through pattern recognition receptors (PRRs), AECs can perceive the presence of Mtb and consequently modulate the composition of the airways surface liquid improving its antimicrobial capacity (16). Moreover, PRRs activation leads to the production of inflammatory cytokines and to the activation of mucosal-associated invariant T cells stimulating IFN-γ and tumor necrosis factor (TNF)-α production (17).

### Macrophages

MΦs are the first line of defense, but only if the ratio of forces lies clearly to their advantage and the intervention is immediate they can cancel the infection (5, 18). Otherwise, they favor its development because they become first a niche for the slow replication of the Mtb and then the sanctuary for the persistence of the infection inside the phagosome during the latent infection phase. Mtb expresses an extremely wide variety of virulence factors that counteract MΦs efforts in suppressing the pathogen. Among Mtb strategies we can include the inhibition of intracellular trafficking, the inhibition of autophagy, the acquisition of cytosol access, the induction of host cell death and the neutralization of toxic components as reactive oxygen species and toxic metals (19).

Whilst IFN-γ is a key element in the containment of Mtb within the MΦ, it is now widely recognized that performing this function requires the presence of vitamin D (63). Thanks to vitamin D, the macrophage increases phagosome maturation and the production of antimicrobial peptides through the maximal regulation of the hCAP-18 gene encoding for cathelicidin antimicrobial peptide which activates, in turn, the transcription of autophagy-related genes (20). The 6 kDa early secretory antigenic target (ESAT-6) protein family secretion (ESX) system is a sophisticated secretion system that Mtb uses to export proteins with immune-escaping activity. So that while the IFN-γ axis is struggling against the ESX-system to enhance phagolysosmal activity, vitamin D deficiency abets the Mtb replication (21).

Nitric oxide (NO) within macrophages plays a less important role in humans than that one observed in animal models (19). Although, in humans too, reactive oxygen species (ROS) play a well-documented role in the immune response to Mtb as highlighted by the discovery of TB susceptibility in patients displaying mutations in a catalytic subunit of NADPH-oxidase 2 involved in ROS production on phagolysosomal membrane (22, 23). Moreover, it is demonstrated that Mtb affects NADPH-oxidase activity through nucleoside diphosphate kinase (Npk) interaction with small GTPases involved in NADPH-oxidase assembly and functioning (24).

The fight unfolds inside the phagosome of the MΦ between the cell and the Mtb with metals as a battlefield of sorts (25). The MΦ delivers an overload of copper and zinc, which are toxic to Mtb at high concentrations. Mtb deploys a series of protection mechanisms that include controlling the capture of such metals, oxidation, and an increase in efflux (25). The up-regulation of *ctpC* gene encoding for the P-type ATPase which regulates the intra-bacterial levels of Zinc is a clear example of how Mtb manages to prevent heavy metal poisoning (26). As a counter-move, the MΦ then attempts to block the arrival of nutrients to the Mtb such as iron and manganese (25).

### Neutrophils

Neutrophil granulocytes are the most widely present cell population within BAL and sputum in patients with active TB (27). There is evidence of their role as defense mechanisms against Mtb. In particular, there is a clear inverse correlation between the number of neutrophilic granulocytes in the peripheral blood and the hazard of developing TB after contact with an infectious subject. Antimicrobial peptides and apoptotic neutrophils are phagocytized by MΦ and carry out an effective activity against Mtb inside these cells (28). This is possible thanks to the fusion, within the MΦ, of neutrophil granules with phagosomes containing Mtb (29). Furthermore, ETosis, extracellular traps (ET) formation, is a type of cell death that differently from apoptosis is characterized by DNA release, consequent MΦ activation and the formation of a DNA scaffold that incorporates pathogens and exposes them to antimicrobial molecules (64). The formation of neutrophils ETs, thus constitutes an improved killing strategy and a synergic alliance between phagocytes.

Moreover, as with many immune mechanisms, neutrophils do not only play a positive role, but can eventually constitute a negative element, causing tissue damage through production and subsequent release of their antimicrobial products (27). To this phenomenon, it must be added the potentially negative interaction with lymphocytes. Neutrophils express on their cell membrane the ligand 1 of cell death (programmed death ligand 1 or PD-L1), which interacts with the lymphocyte receptor for programmed death (programmed death receptor or PD-1), and determines, in the course of chronic infections, the loss of function and finally the death of lymphocytes (30).

Neutrophils with expressed PD-L1 are present in high proportion in patients with ATB.

### Dendritic Cells

DCs are functionally located in the middle between innate and adaptive immunity. These cells play a fundamental role in the immune defense system due to antigen presentation, co-stimulating activity and the large cytokine production capacity with activity on the lymphocytes cluster of differentiation (CD) 4 (32). DCs role in TB immunity is controversial. Present evidence is not sufficient to establish whether these cells strengthen cellular immunity or if their manipulation by the Mtb can be used as a tool to diminish specific T-cell response (31). DCs soon become a niche for the Mtb. CD209, also called DC-specific intercellular adhesion molecule 3-grabbing non-integrin receptor (DC-SIGN), represents the gateway of Mtb into the DC (31). CD209 is, under normal conditions, a receptor for CD54, the intercellular adhesion molecule 1 (ICAM1) present on endothelial cells where it favors DCs migration. CD209 is coupled with the lipoarabinomannan mannose (ManLAM) of the Mtb that penetrates into the cell. This penetration leads to a disruption of DCs activity by prompting the production of interleukin (IL)-10 and reducing the production of IL-12, thus causing a suppression of T lymphocytes activity (33, 34). The manipulation of the maturation of the DCs probably represents one of the winning strategies of Mbt that, by restraining the activity of DCs and, consequently, of T lymphocytes, allows the Mtb, whose speed of growth is relatively slow, to efficiently establish a bridgehead in the airways (35). Based on the above mentioned mechanism DC-SIGN has recently been proposed as a potential target for a vaccine purpose eventually able to enhance immunity against Mtb (36). On the other side, DC-SIGN may prevent tissue pathology by maintaining a balanced inflammatory state and thus promoting host protection (37).

### Natural Killer Cells

It is certain that NK cells enter the immunological circuit of Mtb infection both in their CD56 diminished phenotype (preferential cytotoxic activity) and in CD56 bright phenotype (preferential cytokines secreting activity) (38). In several studies the percentage representation of NK cell is augmented in the peripheral blood of patients with ATB (65). There is a direct relationship between NK cell representation, clinical condition and response to therapy (38, 65). Nonetheless, it has not yet been ascertained exactly what the cause and the consequence is (38).

Several components of the Mbt wall are recognized and bound by the NKp44 receptor of NK cells (39). In addition, Mtb-infected NKs lyse and stimulate MΦs to produce IFN-γ and IL-22, which increase phagolysosomal fusion thus inhibiting Mtb replication and stimulate the production of additional IFN-γ by CD8^+^ lymphocytes. This effect is mediated by the IL-15 and IL-18 production by an infected MΦ. As a further infection control mechanism DCs favor the development of T lymphocytes with γδ receptor through TNF-α and IL-12 production (40).

### Mast Cells

The role of mast cells in Mtb infection is not well-known in humans (41). In mice, mast cells capture Mtb via CD48 and internalize it. This process ensues the development of a cytokine cascade, some of them with protective roles, including IL-12, IL-13, IL-6, CXLL2, CCL7, CCL2, TNF-α, and consequent neutrophils recall in the site of infection (41). Histamine's role is ambivalent in terms of Mtb clearance as on one hand it augments lung neutrophilia but on the other it seems to impair the efficient production of a T helper 1 (Th1) response (42). The presence of mast cell ETs containing Mtb in humans has not been proved. However, mast cells enclose a large number of mediators known to take part in the process (41).

### Complement Proteins

The role of the complement cascade on the progression of the infection and Mtb disease is almost unknown (44). It is likely that the C5 and C7 components play a defensive role. However, it has been observed that a high expression of C1q correlates with a worse clinical condition, so as to be a marker between latent TB and active TB but still with unclear significance in terms of pathogenesis (43, 44).

## Adaptive Immune Response Against *Mycobacterium tuberculosis*

The immune response of T lymphocytes begins at the moment that Mtb spreads inside the lymph nodes but its arousal lays in the early activation of the innate immune system. Inside the lymph nodes, T lymphocytes undergo a process of activation and expansion of the specific populations for the Mtb antigens. However, at this point, the largest part is done and the infection is now established. Cellular immune response can be evidenced 2–6 weeks after Mtb infection by the development of a delayed hypersensitivity response to intradermal injected tuberculin (DHT) or purified protein derivative. It is important to underline that protective response to TB does not relate with DHT positivity and disease can occur in those who mount adequate DHT response (66) (Table 1).

### Lymphocytes T CD4^+^

The *in vivo* human model of HIV-infected CD4^+^-depleted patients is the most striking evidence of the pivotal role of these cells in TB immunity. The process of maturation of the phagosome of MΦ is facilitated and increased by IFN-γ, the production of which is mostly dependent on the T lymphocytes CD4^+^ with a minor support of lymphocytes CD8^+^ and T lymphocytes with γδ receptor (45). Animal models of knockout mice for IFN-γ clearly show that these animals suffer a very severe course of Mtb infection exactly as it happens in humans with MSMD. It is well known that patients with mutations in genes encoding IFN-γ or its receptors undergo disseminated infection by BCG or other non-tuberculous components of the *mycobacteria genus* (12). IFN-γ production is modest in patients with active TB, but recovers with antitubercular treatment without reaching levels similar to those of uninfected subjects.

The optimal production of IFN-γ, as well as that of IL-17 (67), is linked to an equally optimal cooperation between DCs and T lymphocytes CD4^+^. In its defensive strategy, Mtb markedly interferes in the CD40-CD40 ligand binding, that is essential for the cooperation between both cell lines (46). The importance of IFN-γ production by CD4^+^ cells is particularly relevant at the early stages of Mtb infection as it is demonstrated that adequate IFN-γ levels can be obtained with 3 weeks of delay even in CD4-disrupted mice thanks to the compensation offered by other cell types like CD8^+^ (68).

Moreover, IFN-γ cannot control infection alone and it requires the association of other molecules such as IL-6, IL-1 and the TNF-α. The chemokines CCL5, CCL9, CXCL10, and CCL2 attract immunity cells at the site of infection and their production is stimulated by TNF-α and boosts the production of NO by MΦ (47).

Several studies, both in adult and pediatric patients, have demonstrated CD4^+^ percentage and absolute value reduction in the peripheral blood of patients with ATB suggesting both an augmented pooling in the site of infection but also eventually a primary role of TB in immune modifications related with the severity of infection (65).

A portion of T lymphocytes are Foxp3^+^ and perform a control function over the activity of other T lymphocytes in fact, they are defined as T regulators (Treg). It is only on a hypothetical level that we can imagine any positive role of this cell line on the disease progression limiting tissue damage by other immune cells; however, it has been ascertained that, by restraining the response of the T lymphocytes, the Tregs favor the infection development and persistence (48). Similarly, T lymphocytes CD4^+^ may deal more damage, or at least become irrelevant, rather than hinder the progress of the infection (49).

### Lymphocytes T CD8^+^

For a long time, it was considered that, unlike T lymphocytes CD4^+^, T lymphocytes CD8^+^ had no role in controlling the infection and Mtb disease. This concept stemmed from the modest availability of human models with T lymphocyte CD8^+^ defect, unlike the large human model of HIV infection. An activity against Mtb is conceivable considering that T lymphocytes CD8^+^ recognize Mtb antigens through class I molecules of the major histocompatibility complex (MHC), and produce IL-2, IFN-γ and TNF-α, which have a well-known role in controlling Mtb. Furthermore, T lymphocytes CD8^+^ exert a cytolytic action against Mtb by means of perforin and granulysin, albeit not by Fas (CD95) -Fas ligand (50, 51) interaction. This direct cell-to-cell contact determines the apoptosis of the Mtb-infected cell (especially MΦ) depriving Mtb from its natural growth environment and at the same time reducing its viability by unknown mechanism (52). On the other hand, lymphocytes CD8^+^ produce IL-10 and TGF-β which instead favor the development of the Mtb infection.

### Humoral Adaptive Immunity

The role of humoral adaptive immunity in TB is extremely uncertain (53, 54). Complement-mediated opsonization does not alter Mtb survival. High levels of antibody titers correlate with more serious conditions of infection and disease, and passive immunization with antibodies does not confer protection (55). Patients with a defective antibody-production mechanism and/or B lymphocyte defect are not particularly at risk of TB infection.

The role of the crystallizable fragment or Fc in the constant portion of the immunoglobulin, which binds and activates various cell lines present in the granuloma (NK cells, monocytes, neutrophils) the low-affinity FcγRIIIb receptor and the high affinity FcγRIIa receptor have shown different functional profiles and glycosylation patterns in subjects with ATB rather than LTBI (54, 56).

The loss of FcγRIIIb activity and the increase of FcγRIIa-mediated inhibitory function (which correlates with a high IL10 production) are associated with a worse clinical profile and can distinguish ATB from LTBI and suggests a role of antibodies in the augmented phagolysosomal maturation and Mtb killing observed in LTBI patients (56).

### The Ancestral Defense: Granuloma

Following the development of adaptive immunity, a complex and well-coordinated mechanism is established between both immunity mechanisms, i.e., innate and adaptive, which seal the Mtb inside granulomas (5, 58, 59). This mechanism develops in at least 90% of the infected subjects and leads to LTBI. During latent TB, which would be better described as non-replicating-persistence phase (in fact, Mtb works perfectly albeit in a different way than during active TB), the subject is generally positive for the tuberculin skin test and for the IGRA (69). Latent TB becomes active when, for the most various reasons, a condition of immunodepression develops. At this stage, the subject may become capable of transmitting the infection because the granuloma opens in the bronchial lumen and Mtb are expelled when coughing. At the beginning of the infection, Mtb demands an environment with inflammatory traits to develop the granuloma; subsequently, however, its survival is linked to an environment lacking or with low inflammation. This switch is caused by ESAT-6 (60), a well-known Mtb virulence factor involved in the ESX secretion system, to which it gives its name. ESAT-6 causes the transformation of MΦ from phenotype M1, which produces IL-6, IL-12 and TNF-α, into MΦ with phenotype M2, which is capable of stimulating production of IL-10 (60, 61, 70). As currently known, IL-6 and TNF-α favor inflammation, whilst IL-10 curbs it. Accordingly, the formation of the granuloma is triggered by the MΦ and then develops with multi-nucleated giant cells and MΦ with abundant presence of intracytoplasmic lipids, which lend these cells their frothy appearance. Around these cells, there is a ring of T lymphocytes although B lymphocytes, neutrophils and dendritic cells (CD) also participate in the formation of granuloma (4).

Inside the granuloma, cholesterol—and not glucose or glycerol—is the only carbon source. This leads to a lack of carbon and nutrients, hypoxia and a high concentration of nitric oxide (NO). The significance of cholesterol in the survival of Mtb inside the granuloma is evidenced by the negative role that statins play against Mtb (61, 70).

The debate remains open on whether the granuloma is purely protective for the host or if it promotes disease progression and tissue damage (4). This uncertainty depends on the extreme heterogeneity detected in granuloma morphology at the different stages of disease, on the role of inflammation, hypoxia and differential Mtb gene expression and lipid metabolism manipulation inside the granulomas of ATB and LTBI patients (62). The most likely answer is that an homeostatic interaction establishes and the granuloma becomes a well-suited shelter for both Mtb long-term survival and host protection (57).

## Discussion

### *Mycobacterium tuberculosis*: The Great Manipulator

The different cell lines of innate and adaptive immunity come into play at different times in the battle against Mtb in a clash in which the genetic susceptibility of the host and the virulence of the pathogen play decisive roles for the final outcome. The success of Mtb over thousands of years against man arises from its extraordinary ability to subvert the mechanisms that should eliminate it in the MΦ from the infection onset. At the onset of the infection, Mtb manages to perforate the phagosome in the MΦ through the ESX system and, therefore, to block its maturation via Npk, which inhibits lysosomal traffic and NADPH-oxidase activity (31). With various mechanisms, some of which also operate at the level of macrophage DNA, the Mbt prevents the activation of pathogen destruction systems, which are implemented through autophagy. The Mtb DNA manages to prevent the activation of the AIM2 inflammasome thus hindering the synthesis of IL-1β and IL-18 (31). Under normal conditions, IFN-γ stimulates the expression of MHC class II molecules on the MΦ. But Mtb, thanks to the prolonged activation of TLR2, succeeds in suppressing this mechanism. Even in cells that already express class II MHC molecules, Mtb manages to block the presentation of antigens by the action on ESCRT (endosomal sorting complexes required for transport) of its EsxG·EsxH protein (31). The “great manipulator” also interferes with the functions of DCs, neutrophils and all other components of the immune system.

In conclusion, having to deal with a micro-organism of great evasive abilities, immune mechanisms have only one way to go: to focus on a very rapid response at the onset of the infection.

Paraphrasing a famous aphorism by General Erwin Rommel about amphibious battles that is well-suited to TB, victory or defeat against Mtb is decided in the first moments of the infection (5). Exactly as it happened on the first day of the amphibious assault, the day that General Erwin Rommel defined as “the longest day.” When it comes to implementing new prevention and therapeutic approaches, a clear understanding of the interplay between the immune system and Mtb at a molecular level is the only way to unravel this millenary skein.

## Author Contributions

MdM wrote the main body of this minireview. LL, LG, and EC contributed with literature search and revisions.

### Conflict of Interest Statement

The authors declare that the research was conducted in the absence of any commercial or financial relationships that could be construed as a potential conflict of interest. The handling editor declared a past co-authorship with several of the authors MdM, LG, and EC.
